# Postirradiation Sarcoma: Clinicopathologic Features and Role of Chemotherapy in the Treatment Strategy

**DOI:** 10.1155/2009/764379

**Published:** 2009-11-17

**Authors:** Gaetan des Guetz, Alain Chapelier, Véronique Mosseri, Thierry Dorval, Bernard Asselain, Pierre Pouillart

**Affiliations:** ^1^Department of Medical Oncology, Curie Institut, 75005 Paris, France; ^2^Department of Thoracic and Surgery, Marie-Lannelongue Hospital, 92350 Le Plessis-Robinson, France; ^3^Biostatistics Unit, Curie Institut, 75005 Paris, France

## Abstract

*Purpose*. An analysis of the clinicopathologic features and treatment of patients was performed to guide evaluation and management of postirradiation sarcoma. *Patients and Methods*. Between 1994 and 2001, 25 patients with postirradiation sarcoma were treated in one center with different chemotherapy, mainly in neoadjuvant setting (19). Tumors for which these patients received radiotherapy initially were mainly breast carcinoma (for 15 patients). The postirradiation sarcomas were of different histopathologic forms, most frequently osteosarcoma, leiomyosarcoma, and angiosarcoma. *Results*. Of the 25 patients, 19 were initially treated with chemotherapy. Nine of 19 pretreated patients achieved clinical partial response (RP = 47%). Leiomyosarcomas were good responders (3/4) and undifferentiated sarcoma (3/5). Responders were more often treated with MAID (6/8). Eight of the 9 responders underwent surgery. Two patients achieved complete histological response. Seven of the 9 good responders are alive with a median follow up of 24 months. For all treated patients, median follow up 24 months (6–84 months), overall survival and disease free survival were, respectively, 17/25 (68%), and 14/25 (56%). *Conclusion*. From our data, postirradiation sarcoma should not be managed differently from primary sarcoma. Chemotherapy has to be included in the treatment plan of postirradiation sarcoma, in future studies.

## 1. Introduction

Among the treatment options for cancer, radiation therapy (RT) has played an increasingly role, particularly in adjuvant treatment. 

Adverse effects of radiation are sometimes observed, but the most serious is radiation related sarcoma. Since the first report by Beck in 1922, many studies have documented the association between administration of ionizing radiation and the subsequent development of sarcoma [1, 2]. 

Our population was described following the criteria first used by Cahan et al. of postirradiation sarcoma (PIS) [1]:

1. histological confirmation of sarcoma,
2. prior history of RT,
3. latency periods of several years,
4. development of sarcoma within a previously irradiated field.

Surgery is generally considered as the main treatment of PIS. But the poor prognostic of these types of sarcoma, because of the late diagnosis, needs to be evaluated by the association of chemotherapy. Although many reports in the literature concern PIS, there are little data about chemotherapy in the treatment of PIS. Therefore, it would be interesting to explore the role of chemotherapy in the treatment of PIS. In this retrospective analysis, we describe clinicopathological elements and treatment of PIS.

## 2. Patients and Methods

We reviewed the medical records of the patients treated at Institut Curie (IC) and identified in the IC sarcoma treatment register the cases of PIS between 1994 and 2001. Twenty five patients were registered, there were 18 women and 7 men; see Table 1

### 2.1. Initial Tumors Characteristics

There was a predominance of breast cancer (15). The others tumors initially irradiated were limb sarcoma or bone metastases (4), Hodgkin disease (2), and uterin carcinoma (1). In our study we also had one patient with hypophysis adenoma and two adults patients with retinoblastoma. The mean radiation dose delivered was 53 Gy (range from 30 to 72 Gy) with a mean Radiotherapy-sarcoma interval of 12 years (4–36).

### 2.2. Treatments of PIS

The treatment strategy with surgery and chemotherapy was examined. 

There were two approaches to treatment, neoadjuvant or adjuvant setting according to the stage of disease and the possibility of surgical procedures. 

In this neoadjuvant group nineteen patients with locally advanced or metastatic disease were treated with chemotherapy before being evaluated for surgery. 

The chemotherapy consisted usually in 6 cycles of different regimens: cyclophosphamide 500 mg/m^2^, vincristine 1.4 mg/m^2^, doxorubicin 50 mg/m^2^ on day 1, and dacarbazine (DTIC) 400 mg/m^2^ on days 1 to 3 (CYVADIC) cycles repeated every 28 days—the standard regimen was modified after 1998 to MAID, combining, Doxorubicin 60 mg/m^2^ on day 1, ifosfamide 2.5 g/m^2^ on days 1 to 3, and dacarbazine 800 mg/m^2^, given on days 2, cycles repeated every 28 days and combination of Doxorubine 60 mg/m^2^ on day 1, Cisplatine 100 mg/m^2^ on day 1, or ifosfamide 3 g/m^2^ on days 1 to 3 especially for osteosarcoma (Bone regimen). 

The medical treatment was evaluated with clinical and radiological methods (radiography, CT-scann, or MRI) using WHO criteria. 

And it was particularly interesting, for patients who underwent surgical resection to analyse pathologic response (the importance of necrosis on the specimen). This is a valuable parameter of the efficiency of chemotherapy. 

Chemotherapy was also evaluated in terms of cardiac toxicity. 

According to the initial tumor location, thoracic surgery (particularly chest wall surgery and breast surgery) was often performed. Less frequently in our study, surgical resection was also performed on the limbs and the face.

## 3. Statistical Analysis

Followup was calculated from the time of diagnosis of postirradiation sarcoma to the last contact. Actuarial survival curves were plotted from the diagnosis of the radiation related sarcoma using the Kaplan-Meier method. Statistical analysis was based on Fisher's exact test. Outcome of patients after chemotherapy (DFS and OS) was performed.

## 4. Results

### 4.1. Characteristics of Radiation Related Sarcoma

When the PIS was diagnosed, the median age of our patients was 56 years (range from 28 to 68 years). The sites of PIS are clearly linked to the pathology initially treated. So for the 15 women treated for breast cancer, PIS developed in breast and chest wall. Another site of PIS was the leg for the three patients treated initially for bone sarcoma and bone metastasis. The pelvic PIS developed after uterin carcinoma. The others PIS tumors were located in the neck, maxillary, and brain for the patients, respectively, treated for Hodgkin disease, retinoblatoma and hypophysis adenoma. PIS histologic types included osteosarcoma (5), leiomyosarcoma (5), angiosarcoma (4), Schwannosarcoma (2), Malignant Fibrous Histiocytomas (MFH) (2), and undifferentiated or clear cell sarcoma (6). PIS tumors are high-grade sarcomas in this study. 

At diagnosis, twenty one patients had localised tumors; median tumor size is 6 cm (range from 2 to 11) in the neoadjuvant group and 3 cm (2–6) in the adjuvant group. Four patients were metastatic (three in the lungs and one in brain).

### 4.2. Results of Treatments

The 5 patients with osteosarcoma were treated with bone regimen and Cyclophosphamide for 6 cycles and those with soft tissu sarcoma by CyVADic for 6 patients, median number cycles 3 (range 1–4) and MAID (8 patients), median cycles 6 (2–8), according to response rate. Patients with progressive disease usually stopped treatment after 2-3 cycles. 

In the neoadjuvant group of 19 patients initially treated with chemotherapy, before being considered for surgery, nine patients achieved clinical partial response (RP = 47%). Two different histologic subtypes, leiomyosarcoma (4 patients) or MFH, and undifferentiated sarcomas (5 patients) were frequently characterised. We observed higher response rate for these tumours, respectively, 3/4 (75%) and 3/5 (60%). This was rather different for other subtypes as osteosarcoma 1/4 (25%), angiosarcoma 1/3 (30%), schwannosarcoma 1/2 (50%), or chondrosarcoma 0/1. Moreover with chemotherapy as MAID we have higher response rate, 6/8 (75%) compared to Cyvadic, 2/6 (30%). For leiomyosarcoma, two of the 3 responders have been treated by MAID. 

For the nine clinical responders, eight patients could be operated on. The sarcomas which were removed after chemotherapy were examined histologically for the presence of residual tumor. Pathological analysis found two complete histological responses. 

These characteristics of patients and results of treatments are summarised in Table 1.

Different types of surgery were performed for all the patients who were operated on. According to the sites, mainly breast and chest wall surgery for five patients, there are two limb surgeries and one maxillary surgery after retinoblastoma. One patient with lung metastases was operated on, he was treated with double surgery, for sarcoma and lung metastases. Moreover chest wall reconstruction was often necessary in all the cases of major treatment. The margins were not involved in these cases. 

One patient had tumor located in the brain (after hypophysis adenoma). Even with objective response, this tumor could not be removed because of sinus cavernous involvement.

### 4.3. Followup and Outcome

For all treated patients, the median followup reached 24 months (6–84 months). 

**Figure 1:**
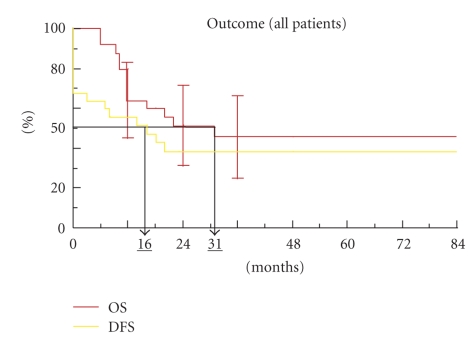
Overall survival and disease-free survival (OS, DFS).

The median overall survival (OS) was 31 months and disease-free survival (DFS) was 16 months for all the patients (Figure 1). 

For the 19 patients in the neoadjuvant group, median OS was 20 months. In the neoadjuvant group, we could differentiate survival differences between patients treated only with chemotherapy and those who also underwent surgery. Even with this low number of patients, the differences are significant; *P* = .0009. Therefore survival was much better in patients who presented clinical response and who were operated on. It seems not possible to differentiate prognostic according to histology, with the many different types of sarcomas in this small series. 

On the other hand, in the adjuvant group, 1/6 (17%) patient developed recurrent disease. For these patients, the median OS was not reached. 

One patient died, not from disease progression but from heart failure. We also have to note that the cardiotoxicities were observed for 2 patients showing clinical signs (grade 3-4 of New York Heart Association (NYHA) criteria). However the rate of LVEF could be normalized for all these patients except one.

## 5. Discussion

These sarcomas are rare, Mark's data estimated the risk of PIS to be from 0.03 to 0.8% with a long-term followup (fifteen years) [3]. Thus given the large number of patients who can be cured or receiving adjuvant RT, we have to consider that the total number of patients who develop PIS is not so small. Ewertz and Mouridsen estimated a RR of 2.3 for bone sarcoma and 2.1 for connective tissue sarcoma (*P* < .5) [4]. 

Particular types of tumors are retinoblastoma. These patients treated for retinoblatoma with deletion of the retinoblastoma gene, an antioncogene, which also contribute to the second primary malignancies, have increased risks of sarcomas. So patients treated for retinoblastoma are a specific population but we included two patients because they were treated as others PIS cases [5]. 

The frequency of breast cancer and radiotherapy for the treatment of this pathology explains the PIS female predominance. So in our study, most of the patients were treated for PIS after breast cancers (12 cases). 

About pathologic type of PIS, there were 4 bone sarcomas and 21 soft tissue sarcomas. Osteosarcoma is frequently observed; the other subtypes are leiomyosarcoma, HFM or fibrosarcoma, and schwannoma but it is difficult to differentiate the prognosis of these subtypes [6]. 

As outlined in the previous studies, surgery appears to play an important role in the improvement of survival for PISs [6–10]. According to Pitcher, the only long-term survivors were those who had complete surgical resection [7]. Radical resection of the tumor is the only chance of cure regardless of location, although centrally located PIS had a worse prognosis than those developed in the limbs which could be amputated or resected [8–10]. So in our study, the majority of patients were treated with surgery, which could explain the slightly better survival rate. 

So in our study, the majority of patients were treated with surgery, which could explain the slightly better survival rate compared with other published studies. Women who underwent aggressive surgery (amputation or chest wall resection) for PIS had a favorable outcome, therefore future patients may benefit from similar management). Because secondary sarcomas arise in irradiated areas, surgical procedures are often difficult. Therefore, patients who develop RIS should be evaluated at an institution which has extensive multidisciplinary experience with RIS (radiotherapy, surgery, and chemotherapy) [11]. 

The success of surgical resection is also due to the organs involved. For example in the brain, the surgical treatment was not possible in our case after response to chemotherapy. The possibility of surgical resection also depends on the initial stage. For the four initially metastatic patients, only one was operated on. In the Brady study only including patients whose primary tumors were in the breast and in whom surgery had been performed also found interesting good results (30% survival at 5 years) [12]. 

The interest of this study is to consider chemotherapy for the treatment of these types of sarcomas. Such tumors generally are aggressive and have a high potential for local recurrence and for metastases. Chemotherapy treatment is logical because of the possibility of dissemination of disease for the voluminous and particularly high histopathologic grade of these sarcomas. So for the patients treated with chemotherapy we obtain encouraging results. Neoadjuvant treatment has the double benefit, to evaluate chemosensitivity and to begin chemotherapy without delay providing a surgical resection sometimes not possible initially. According to many authors, chemotherapy is rarely proposed or not considered as the main treatment for PIS. Few reports are interested in the role of chemotherapy in PIS [13], except for the case of osteosarcomas which are known as being particularly sensitive to the chemotherapy. Cefalo related a study with five children treated for PIS osteosarcoma with a chemotherapy protocol similar to that used in cases of primary osteogenic sarcoma. Four patients were still living 1 to 12 years after treatment [14, 15]. We also observed good response rate especially for leiomyosarcoma or undifferentiated soft tissues sarcomas. Chemotherapy could be proposed for PIS as neoadjuvant treatment to reduce the surgical intervention. It seems particularly interesting to note that the response rate was higher (OR = 47%). Moreover, in some cases the chemotherapy allows us to observe response and so for these cases to permit later surgical resection. Considering the better response rate for MAID regimen, it seems especially benefit for patients who could be considered for surgery to propose this type of treatment. 

A few patients who were treated with neoadjuvant or adjuvant chemotherapy are noted as long survivors. When we examined protocols of chemotherapy based on adriamycin, it is necessary to note the cardiac toxicity. The heart is particularly vulnerable after breast cancer treatment. Several reasons could explain this type of toxicity, the initial chemotherapy for the primary tumor, often containing anthracycline and the retreatment for secondary sarcoma and the possible consequences of radiotherapy for breast cancer irradiated. And so we have to mention that the patients are generally older and have other vascular risk factor. So it would be a necessity to prevent and follow the development of cardiotoxicity [16]. 

The prognosis in patients with PIS is generally poor; median survival rate of PIS is one year [5]. In our study the median OS appears to be better with a median survival rate of 31 months. It seems necessary to compare with others primaries sarcomas.

In the studies regarding sarcoma, prognostic factors such as tumor size, location, surgical resection and chemosensitivity are known, especially with osteosarcoma [17]. Factors as size and site of these tumors can explain the poor prognosis in patients with secondary sarcoma. PIS is often difficult to diagnose for patients with fibrosis lesions after radiotherapy. So the diagnosis is late and the tumor size is larger. Therefore, for PIS and sarcoma, the prognostic factors would be considered as the same. 

To conclude, a strategy based on chemotherapy and surgery is proposed, and the patients who benefited from complete surgical resection had the best prognosis. The chemotherapy can be proposed as part of the strategy in these patients. High survival rates were observed for patients treated with surgery plus chemotherapy. PIS should be treated in the same way as sporadic sarcomas. 

Further studies should be conducted in this field to determine at which stage chemotherapy is most beneficial, either as neoadjuvant, adjuvant, or both, taking into account histologic features, grade type, and location of the tumor.

## Figures and Tables

**Table 1:** Characteristics of patients in neoadjuvant group.

Clinicopathologic factor	Patients
Age (median, years)	56 years

Sex	
Male	7
Female	18

Primary tumour	
Breast cancer	15
Hodgkin disease	2
Bone metastase	1
Sarcoma	
– Ewing sarcoma	2
– Osteosarcoma	1
Retinoblastomas	2
Uterus carcinoma	1
Hypophysis adenoma	1

Radiotherapy, dose delivered (mean, grays)	53

Time to development (range)	12 years (4–36)

Size, mean cm, (range)	6 (2–11)
For adjuvant group	4 (2–4)
Neoadjuvant group	6 (2–11)

Presentation for all patients:	
Local disease	21
Metastatic disease: lung	2
Brain	1

Pathologic characteristics for neoadjuvant group	
(response rate)	
Leiomyosarcoma	4 (3/4)
Osteosarcoma	4 (1/4)
Angiosarcoma	3 (1/3)
Schwannosarcoma	2 (1/2)
Chondrosarcoma	1 (0/1)
MFH and sarcoma undifferentiated	5 (3/5)

Neoadjuvant chemotherapy	
(response rate)	
– CYVADIC	(2/6)
– MAID	(6/8)
– Doxo-CDDP	5 (1/5)

Adjuvant chemotherapy	
– CYVADIC	1
– MAID	1
– Doxo-CDDP/ifosfamide	4

MFH: malignant fibrous histiocytomas.
